# Unlocking the value of RSV vaccination in Japan: a return on investment analysis using an integrated actuarial-macroeconomic model

**DOI:** 10.3389/fpubh.2026.1871916

**Published:** 2026-08-04

**Authors:** Eleftherios Zarkadoulas, Asako Akimoto, Deven Chauhan, Yufan Ho, Christianah Kolajo, Nikolaos Kotsopoulos, Kentaro Tajima

**Affiliations:** 1 GSK, Wavre, Belgium; 2 GSK, Tokyo, Japan; 3 GSK, London, United Kingdom; 4 GSK, Singapore, Singapore; 5 GSK, Rockville, MD, United States; 6 Health Economics, Global Market Access Solutions Sàrl, Chardonne, Switzerland; 7 University of Athens, Athens, Greece

**Keywords:** actuarial-macroeconomic model, cost–benefit analysis, Japan, older adults, respiratory syncytial virus, return on investment, RSV prefusion F protein vaccine, societal value

## Abstract

**Objective:**

Respiratory syncytial virus (RSV) causes significant morbidity and mortality among older adults and individuals with chronic conditions. This study conducted a cost–benefit analysis (CBA) to evaluate the societal value of RSV vaccination with the adjuvanted RSV prefusion F protein (RSVPreF3) vaccine among older adults aged ≥60 years and high-risk adults aged 50–59 years in Japan.

**Methods:**

An actuarial population projection model, combined with a full CBA framework adapted to the Japanese setting, was used to estimate costs and benefits of adjuvanted RSVPreF3 vaccination versus no vaccination over a 10-year time horizon. Deterministic sensitivity analyses were conducted to assess parameter uncertainty.

**Results:**

Our results indicate that a 5-year vaccination program among older adults, including those aged 50–59 years at higher risk for severe RSV disease and those aged ≥60 years, was associated with substantial economic benefits. Among older adults aged ≥60 years, the total return on investment (ROI) was 3.3 with or without deadweight loss (DWL), with vaccination projected to avert Japanese Yen (JPY) 35,501 M in productive output loss, save JPY 1,178,403 M in direct healthcare costs, and prevent JPY 10,699,729 M in monetized quality-adjusted life-year (QALY) loss. For high-risk adults aged 50–59 years, the total ROI was 3.7 without DWL and 3.8 with DWL, with corresponding reductions of JPY 17,889 M in productive output loss, JPY 10,400 M in healthcare costs, and JPY 266,116 M in monetized QALY loss.

**Conclusion:**

Overall, adjuvanted RSVPreF3 vaccination among older adults in Japan represents good value for money from a societal perspective, where vaccine acquisition and administration costs are expected to be outweighed by substantial health, productivity, and broader economic benefits.

## 1 Introduction

Respiratory syncytial virus (RSV) is a highly contagious virus that causes acute respiratory infections (ARI), with clinical manifestations ranging from upper respiratory tract disease (URTD) with mild respiratory symptoms, to severe and potentially life-threatening lower respiratory tract disease (LRTD) (1–3).

Although RSV infection can occur across all age groups, severe outcomes among adults are predominantly observed in those who are older and have underlying conditions (4–10). In older adults, immunosenescence and comorbidities heighten susceptibility to infection and contribute to more severe disease, leading to substantial healthcare resource utilization (HCRU) (4–6, 8, 9). Individuals with chronic underlying conditions (e.g., asthma, chronic obstructive pulmonary disease [COPD], metabolic conditions [e.g., diabetes], chronic kidney disease [CKD], and cardiovascular disease [CVD]) are also at elevated risk, as these conditions are known risk factors that increase vulnerability to RSV infection and its complications (5–8, 10). Moreover, RSV infection can exacerbate pre-existing comorbidities, further amplifying the overall disease burden (7).

In Japan, RSV infection imposes a considerable healthcare and economic burden, particularly among older adults and those with comorbidities (6, 11, 12). Among Japanese older adults, RSV has been identified as one of the leading causes of ARI. In a prospective study, RSV was detected in 2.4% of adults aged ≥65 years with acute respiratory disease (13), while a separate multicenter study of outpatient adults aged ≥60 years presenting with ARI reported RSV prevalence estimates of 2.5 and 2.8% in two separate RSV seasons (11). Affected individuals often experience functional decline and reduced health-related quality of life (HRQoL) (11). RSV infection among older adults further imposes a substantial impact on medical costs, where an analysis of health insurance claims data in Japanese older adults aged ≥60 years showed that direct medical costs increased by approximately Japanese Yen (JPY) 58,000–234,000 within 30 days after an RSV event compared with the pre-event period (14). Additionally, for adults with underlying conditions, a systematic review of data from industrialized countries reported 1.3-fold higher rates of RSV-related hospitalization and 5.3-fold higher rates of intensive care unit admission than in older adults aged ≥60 years without such conditions (6).

Given the absence of RSV-specific antiviral therapies, vaccination represents an effective strategy to prevent disease onset and reduce the associated clinical and economic burden. Currently, three types of RSV vaccines – the adjuvanted RSV prefusion F protein (RSVPreF3) vaccine, bivalent RSV prefusion F protein-based (bivalent RSVPreF) vaccine, and mRNA-based mRNA-1345 vaccine – are approved for older adults aged ≥60 years in Japan (15–18). The adjuvanted RSVPreF3 vaccine was first approved in Japan in September 2023 for RSV disease prevention among older adults aged ≥60 years based on data from the AReSVi-006 phase 3 clinical trial (NCT04886596) (15, 17), where the vaccine showed statistically significant and clinically meaningful overall efficacy of 82.6% against RSV-LRTD, and demonstrated an acceptable safety profile in this population (19, 20). In November 2024, the indication was expanded to include adults aged 50–59 years who are at increased risk of severe RSV disease. This was supported by a trial that demonstrated the non-inferior immunogenicity and consistent safety profile of the adjuvanted RSVPreF3 vaccine in adults aged 50–59 years, compared with those aged ≥60 years (21, 22). Additionally, a recent cost-effectiveness analysis (CEA) incorporating up-to-date clinical data over three full RSV seasons reported incremental cost-effectiveness ratios (ICERs) of JPY 2,770,558 per quality-adjusted life-year (QALY) gained in high-risk adults aged 50–59 years and JPY 2,613,241 per QALY in older adults aged ≥60 years (23). This further demonstrated the robust cost-effectiveness of the adjuvanted RSVPreF3 vaccine.

In Japan, vaccines are classified under the National Immunization Program (NIP) as either routine or voluntary, in accordance with the Immunization Act. Routine immunizations are implemented by municipalities and include Category A diseases, which prioritize collective prevention and are fully funded (24). In contrast, voluntary immunizations for Category B diseases, such as seasonal influenza and COVID-19, emphasize individual prevention and are only partially funded, resulting in out-of-pocket costs for individuals (24). Despite the growing body of evidence demonstrating the substantial burden of RSV disease in Japanese older adults, as well as the safety, efficacy, and cost effectiveness of RSV vaccines, an NIP for RSV disease prevention in older adults has not been introduced (25). The absence of an NIP limits patient access to the adjuvanted RSVPreF3 vaccine and represents a key barrier to uptake, likely due to vaccine costs in the absence of public funding (23, 26). Therefore, the introduction of an NIP for RSV disease prevention in older adults should be regarded as a necessary initial measure to reduce financial barriers and improve access and uptake.

Robust economic evidence is crucial for supporting the introduction of an NIP for RSV disease prevention in older adults, and can also help to raise awareness of RSV’s broader societal burden. Although CEAs provide essential evidence for assessing value for money, they do not capture the broader economic returns that vaccination may generate for healthcare systems and society, such as Gross Domestic Product (GDP) gains and other macroeconomic benefits. Similar cost–benefit analyses (CBA) conducted for other vaccination programs have shown that evaluating these wider impacts can be critical for informing national immunization policy (27–30). This is particularly relevant in Japan, a super-aged society projected to have one in 2.6 people aged ≥65 years and one in around 4.0 people aged ≥75 years by 2070 (31). The mitigation of productivity losses and sustainability of public finances are key economic policy priorities, given rising employment rates among those aged ≥65 years (31). Building on this precedent, there is a critical need to comprehensively evaluate the broader economic value of the adjuvanted RSVPreF3 vaccine in Japan, as these healthcare system and societal level gains remain insufficiently explored.

Therefore, this study aimed to quantitatively assess the long-term sustainability and economic feasibility of investing in RSV vaccination for older adults aged ≥60 years and high-risk adults aged 50–59 years in Japan, among whom comorbidities such as CVD and diabetes are prevalent (32). To address this, we conducted a broader CBA that monetized key public health outcomes (e.g., medical cost savings, avoided productivity losses, and averted QALY reductions) to assess the overall benefits and costs of RSV vaccination with the adjuvanted RSVPreF3 vaccine among Japanese older adults aged ≥60 years and those aged 50–59 years at higher risk for severe RSV disease.

## 2 Methods

### 2.1 Analysis specifications

#### 2.1.1 Population

This analysis focused on two target populations: older adults aged ≥60 years and adults aged 50–59 years who are at an increased risk of severe RSV disease due to certain underlying diseases (hereafter referred to as high-risk adults aged 50–59 years), including asthma, CKD, chronic liver disease (chronic hepatitis and cirrhosis), COPD, congestive heart failure (coronary artery disease and heart failure), and diabetes (type I, type II, and other) (5, 33–35). These selected diseases were based on those analyzed in the RSV OA = ADJ-018 trial (NCT05590403) that evaluated adjuvanted RSVPreF3 immunogenicity in high-risk adults aged 50–59 years at increased risk of severe RSV disease due to underlying medical conditions (21).

Population sizes were obtained from population estimates published by the Official Statistics in Japan (36). As individuals in the high-risk 50–59 years population were categorized by primary diagnosis, the diagnosis-specific subpopulations were mutually exclusive and additive in deriving the overall population of high-risk adults aged 50–59 years.

#### 2.1.2 Time horizon

Vaccination was assumed to occur over the first 5 years. During each year of the 5-year vaccination period, the initial population within the specified age cohort were vaccinated, alongside successive new cohorts of adults reaching the target age of the cohort analyzed. For instance, the analysis for the 50-year-old cohort included the initial population aged 50 years in Year 1, and new 50-year-olds entering in Years 2 through 5. Each age cohort (i.e., individuals aged 50 years and above through to 100 years) was modeled independently, with the analysis repeated separately for each cohort. Vaccination was assumed to be administered as a single, one-time dose to eligible individuals within each cohort in scope, based on age-specific vaccine coverage assumptions consistent with influenza vaccine uptake reported by the National Institute of Infectious Diseases (37). No revaccination was considered.

The model used a 10-year analytical time horizon to capture the benefits of averted morbidity, including all RSV cases and associated costs averted under vaccination. This allowed the last vaccinated cohort at Year 5 to be followed up in a consistent manner, along with the vaccine’s waning and duration of protection, thus capturing the impact of vaccination. A lifetime time horizon was used to assess QALYs lost due to RSV-attributable deaths, capturing the benefits of preventing mortality.

#### 2.1.3 Discount rate

An annual discount rate of 2% was applied to both costs and QALYs, in accordance with the Japanese economic evaluation guidelines and consistent with the previous CEA (23, 38, 39).

### 2.2 Model details

This study employed an open population projection model combined with a societal CBA framework – integrating direct medical healthcare costs borne by the healthcare system, productivity losses, QALY monetization, and deadweight losses – to estimate the overall benefits and costs of adjuvanted RSVPreF3 vaccination.

#### 2.2.1 Actuarial method for population projections

An actuarial population projection approach, consistent with International Labor Organization (ILO) methods (40), was used to dynamically simulate demographic changes over time. This approach draws on national statistical data and accounts for key components of population change, such as mortality. Gender and net immigration were excluded from this actuarial analysis, given that population-level RSV outcomes (e.g., cases prevented and economic impact) are driven primarily by age and risk status (41). The population was divided into single-year age cohorts and categorized into status groups of employed, disabled, retired, and dead. Cohorts were moved forward annually and updated using age-specific mortality, disability, and retirement rates, with new entrants added each year as cohorts aged (i.e., introduction of individuals aged 50 years, who originated from the previous age group [i.e., 49 years] after adjusting for mortality). Individuals newly reaching 50 years were vaccinated according to assumed coverage levels. Labor force projections were calculated by applying age-specific labor force participation rates to the projected cohorts and updating each cohort’s composition as they age. Employment projections were similarly updated each year across all age groups over the 10-year time horizon, allowing employment outcomes to be modeled dynamically.

#### 2.2.2 Disease simulation model

Epidemiology and RSV natural history projections produced by the disease simulation model were aligned with that produced by previous adjuvanted RSVPreF3 vaccine older adult cost-effectiveness models (23, 42, 43). The model used annual cycles and three health states – no RSV, post-RSV, and RSV death – to project RSV epidemiology, with all-cause mortality (i.e., non-RSV specific) applied to the first two states. At the start of the simulation, the entire population was in the ‘no RSV’ health state. RSV epidemiology was modeled through transitions representing RSV, URTD, LRTD, and complications, assuming no reinfection within the same annual cycle. For both RSV vaccination and no-vaccination scenarios, the model estimated number of RSV cases, while direct medical costs and QALYs were calculated based on these health and transition states, with work absenteeism estimated based on RSV-related events. Vaccine waning was also incorporated to account for the reduction in protection over time.

#### 2.2.3 Model inputs

Model inputs drawn from a previously published CEA included epidemiological parameters (e.g., RSV infection incidence, proportion of LRTD cases, and RSV-related mortality; Supplementary Table S1), vaccine parameters (e.g., efficacy, age-specific coverage rate, and costs; Supplementary Table S2), HCRU (e.g., frequency of hospitalizations and outpatient visits) and unit costs, based on which direct medical costs were calculated (Supplementary Table S3), and HRQoL inputs (e.g., age-specific loss of QALYs per RSV-related event and baseline utility values; Supplementary Table S4) (23). Demographic and actuarial inputs (Supplementary Table S5) were sourced from national official statistics. Key economic inputs are summarized in Supplementary Table S6, where estimated costs were also adjusted for inflation, GDP growth, and wage growth to account for real-world economic changes.

### 2.3 Broader economic analysis

#### 2.3.1 Analytic perspective

The broader economic evaluation was conducted as a CBA from a societal perspective. The analysis was informed by the estimation of RSV-related outcomes under vaccination and no-vaccination scenarios. These outcomes included URTD and LRTD cases, HCRU, RSV-attributable deaths, and QALYs lost due to RSV.

The primary economic outcome produced in the CBA was the return on investment (ROI), which compared the present value of vaccination benefits with vaccination costs, under the societal perspective. Societal gains were assessed in terms of healthcare cost savings in direct medical costs, averted productive output loss in the economy, and averted monetized QALY loss. Direct healthcare cost savings and monetized QALY losses represent distinct and non-overlapping benefit categories. The former captures reductions in medical resource utilization attributable to vaccination, while the latter reflects the welfare value of averted health losses—including pain, functional decline, and premature mortality—that are not accounted for in direct cost estimates. Both components were included under a societal perspective and were summed without double-counting. Costs of investment were represented by vaccination administration and acquisition costs for the vaccination cohort.

#### 2.3.2 Vaccination costs

Vaccine acquisition and administration costs were applied using age-specific coverage assumptions. Vaccination was assumed to occur in the first 5 years of the 10-year time horizon, with annual vaccination of each cohort entering the study during this period.

#### 2.3.3 Direct medical costs

Direct medical costs were consistent with the previous CEA model and included disease management costs, i.e., inpatient and outpatient costs (23). These were calculated by applying local unit costs to HCRU associated with RSV-related events, such as URTD, LRTD, and hospitalizations.

#### 2.3.4 Productivity loss and QALY monetization

Vaccination reduces the impact of RSV and its complications, thereby preventing RSV-related absenteeism and subsequent loss of productive output by the economically active and employed individuals experiencing RSV. RSV-related outcomes, i.e., URTD and LRTD requiring outpatient and/or inpatient care were associated with specific workhour losses (Supplementary Table S6), which were then monetized to reflect the economic impact of RSV-related absenteeism. This analysis employed the Cobb–Douglas production function to estimate the resulting GDP losses. The function is typically expressed as follows:


$$Y=A\cdot K^{\alpha }\cdot L^{\beta }$$

Where *Y* denotes total output (i.e., GDP in market prices [JPY]); *A* represents total factor productivity (TFP), reflecting the efficiency with which capital (*K*) and labor (*L*, measured as total hours worked) are utilized; and *α* and *β* are the output elasticities of capital and labor, respectively.

This assumes that productivity output, i.e., GDP is a function of capital and labor, and accounts for the capacity of capital to partially compensate for reduced labor input, thereby yielding a more conservative estimate of productivity loss. Using the labor elasticity parameter (*β*) where the contribution of labor to GDP was 50% (44), GDP loss was calculated as follows and translated into loss of productive output.


$$\begin{array}{l} \text{Total}\;\mathrm{GDP}\;\text{Loss in year}\;t:\mathrm{YL}(t)=\\ \mathrm{GDP}\;\text{in year}\;t\;(t)\times \frac{\text{Total work hours loss from}\;\mathrm{RS}V_{\text{year}\;t}\times \beta }{\text{Total hours worke}d_{\text{year}\;t}} \end{array}$$

The loss of 1 monetary unit of GDP is expected to result in loss of tax revenue, since a fraction of GDP returns to the government in the form of tax. This analysis quantified the tax revenue loss as a subproduct of the GDP loss estimated in the aforementioned societal analysis. GDP losses (*YL[t]*) due to RSV-related absenteeism were also translated into tax revenue losses. Specifically, GDP loss was used to estimate loss of tax revenues using the percentage of GDP that corresponds to payments of social insurance and taxes. Hence, the following estimates were generated: social insurance contributions (social insurance loss[*t*] = *YL[t]* * % social insurance), direct tax, e.g., income tax (direct tax loss[*t*] = *YL[t]* * % direct income tax), indirect tax, e.g., consumption tax (indirect tax loss[*t*] = *YL[t]* * % VAT indirect tax), and corporate tax (corporate tax loss[*t*] = *YL[t]* * % corporate tax).

In addition to morbidity-related absenteeism, productive life-years lost due to premature RSV mortality were calculated and monetized by adjusting remaining life expectancy. GDP per capita was selected as the base-case value for monetizing QALY losses, consistent with its established use in public health ROI analyses as a proxy for the value of a statistical life-year. This metric also approximates, in broad terms, the purchasing power lost due to disease burden. In the absence of a formally adopted QALY monetization threshold for evaluating the ROI of broader public health infrastructure and preventive programs in Japan, GDP per capita represents a conservative and internationally comparable reference value. To assess robustness, higher valuations (2 × and 3 × GDP per capita) for QALY monetization were also explored in scenario analyses. All costs were converted into JPY in 2024 prices.

#### 2.3.5 Deadweight loss (DWL)

DWL was calculated based on tax revenue losses to reflect the economic inefficiency associated with taxation, recognizing that taxes and subsidies are not cost-free. In Japan, the marginal costs of increasing labor tax was assumed to be equivalent to that in the European Union (EU) at 0.79 (45).

#### 2.3.6 Economic appraisal indices

The main index produced was the ROI, which compares the broader economic gains of vaccination with its costs. ROI is the ratio of the present values of benefits divided by the present values of costs subtracted by 1, where an ROI > 0 indicates that benefits outweigh costs, resulting in a profitable intervention that generates positive economic return. In this analysis, benefits refer to averted GDP loss from avoided absenteeism, health costs-savings, and monetized averted QALY loss, while costs refer to vaccine acquisition and administration costs. ROI was calculated using the following equation:


$$\mathrm{ROI}=\frac{\text{Present value of benefits}}{\text{Present value of vaccination costs}}-1$$

Financial analysis indicators included the internal rate of return (IRR), which reflects the average annual return on the vaccination investment, as well as the payback period, which describes the time required for benefits to outweigh costs.

### 2.4 Sensitivity and scenario analyses

#### 2.4.1 Sensitivity analyses

Given the deterministic structure of the study and its focus on estimating net returns per JPY invested in RSVPreF3 vaccination by evaluating its overall benefits and costs, a probabilistic sensitivity analysis was not conducted. Instead, uncertainty was explored through extensive one-way deterministic sensitivity analyses (DSA) across key parameters to assess their individual impact on the estimated ROI, by varying model inputs one at a time across ranges. Model inputs were varied to test the robustness of the results and the significance of each factor. Results of the DSA are presented in a tornado diagram.

#### 2.4.2 Scenario analyses

Scenario analyses using higher thresholds to monetize QALY (e.g., 2 × or 3 × GDP per capita, the value of statistical life year [VSLY], or a cost-effectiveness threshold per QALY) were conducted to explore the robustness of results. A cost-effectiveness threshold of JPY 5.5 M was assumed, as an ICER of less than JPY 5–6 M per QALY gained is generally considered to be cost-effective (46). The VSLY (JPY 15 M) was calculated by annualizing the value of statistical life (VSL; i.e., dividing VSL by 40 years) where the VSL was assumed to be JPY 600 M, representing the lower range of the reported VSL for Japan (47).

A financial analysis was also conducted by simulating a single birth cohort each time, without allowing additional cohorts to enter the model. The IRR and payback period were estimated for this fixed cohort.

## 3 Results

### 3.1 Base case analysis (older adults aged ≥60 years)

Over a 10-year time horizon, the 5-year vaccination program with the adjuvanted RSVPreF3 vaccine in older adults aged ≥60 years was estimated to avert a productive output loss of JPY 35,501 M due to RSV-related absenteeism, save direct healthcare costs of JPY 1,178,403 M, and avoid a QALY loss of JPY 10,699,729 M due to mortality and morbidity (Table 1). The total costs from vaccine acquisition and administration were estimated at JPY 2,776,561 M. Overall, the total ROI for older adults aged ≥60 years was 3.3 without or with DWL.

**Table 1:** Primary analysis of base case scenario.

Age	Averted productive output loss	Healthcare costs-savings	Vaccine costs	QALY gains monetized	Societal ROI without DWL	Societal ROI with DWL
50–59 (HR)	17,889 M	10,400 M	62,173 M	266,116 M	3.7	3.8
60–85	35,501 M	1,178,403 M	2,776,561 M	10,699,729 M	3.3	3.3

Societal benefit encompasses averted productive output losses, direct healthcare cost savings and monetized QALY gains, and respective data are presented separately. Monetary values appear in JPY, 2024. DWL: deadweight loss; HR: high-risk adults at increased risk of severe RSV disease due to certain underlying diseases; QALY: quality-adjusted life years; ROI: return on investment.

### 3.2 Base case analysis (high-risk adults aged 50–59 years)

Over a 10-year time horizon, the 5-year vaccination program with the adjuvanted RSVPreF3 vaccine in high-risk adults aged 50–59 years was estimated to avert a productive output loss of JPY 17,889 M due to RSV-related absenteeism, save direct healthcare costs of JPY 10,400 M, and avoid a QALY loss of JPY 266,116 M due to mortality and morbidity (Table 1). The total costs from vaccine acquisition and administration were estimated at JPY 62,173 M. Overall, the total ROI for high-risk adults aged 50–59 years was 3.7 without DWL and 3.8 with DWL.

### 3.3 Sensitivity analyses

**Figure 1:**
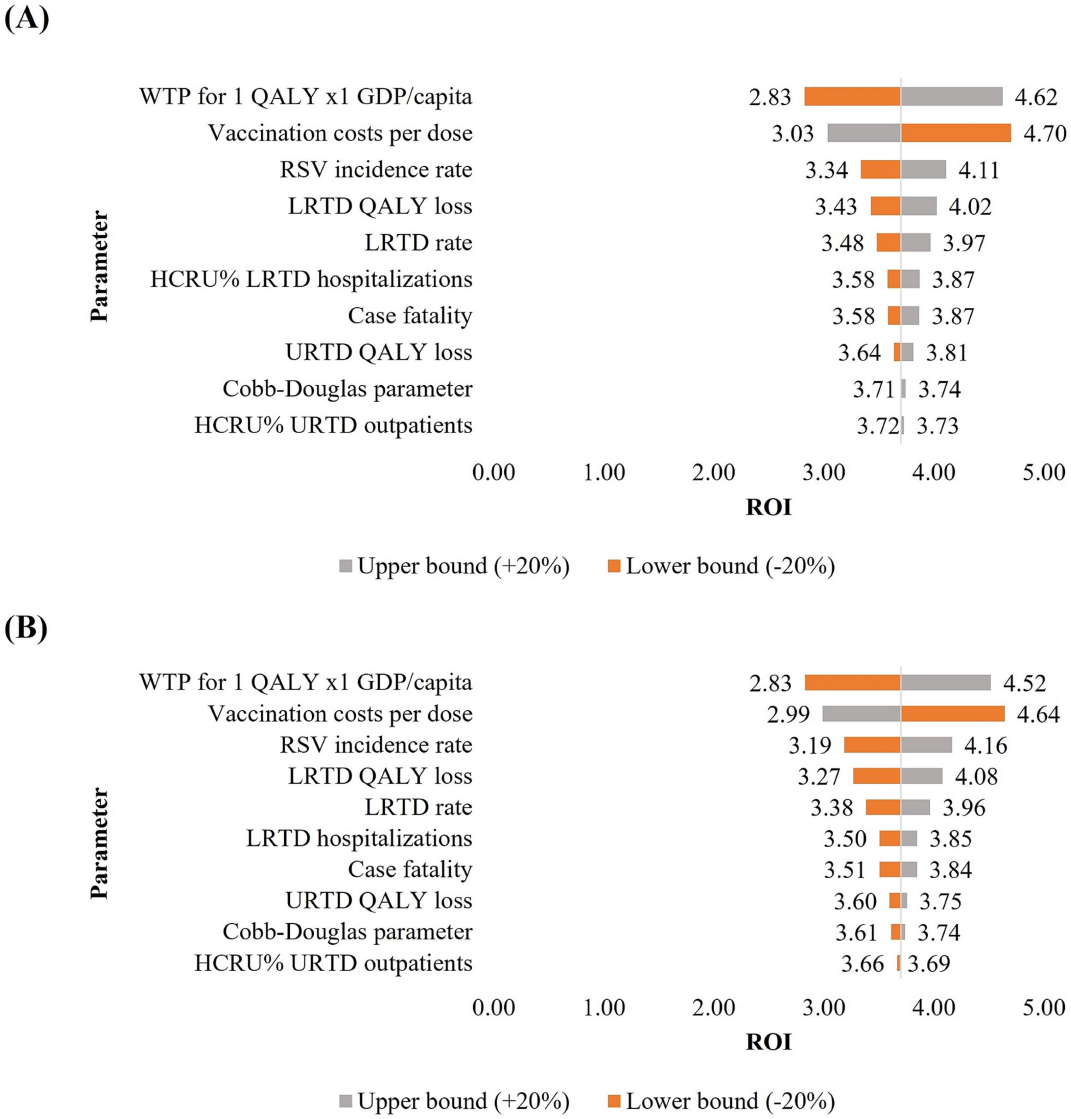
Deterministic sensitivity analysis of base case scenario. **(A)** Consecutive cohorts of older adults aged 60 years; **(B)** Consecutive cohorts of high-risk adults aged 50 years. GDP: gross domestic product; HCRU: healthcare resource utilization; LRTD: lower respiratory tract disease; QALYs: quality-adjusted life years; ROI: return on investment; RSV: respiratory syncytial virus; URTD: upper respiratory tract disease; WTP: willingness-to-pay.

In consecutive cohorts of older adults aged 60 years, societal ROI was the most sensitive to the willingness-to-pay threshold for 1 QALY valued at 1 × GDP per capita, followed by vaccination costs per dose, and RSV incidence rate. The highest societal ROI observed was 4.7 when vaccination costs per dose were varied to its lower bound (Figure 1A).

In consecutive high-risk cohorts of adults aged 50 years, societal ROI was similarly the most sensitive to the willingness-to-pay threshold for 1 QALY valued at 1 × GDP per capita, followed by vaccination costs per dose, and RSV incidence rate. The highest societal ROI observed was 4.6 when vaccination costs per dose were varied to its lower bound (Figure 1B).

### 3.4 Scenario analyses

#### 3.4.1 Societal ROI under different assumptions for the QALY monetization

**Figure 2:**
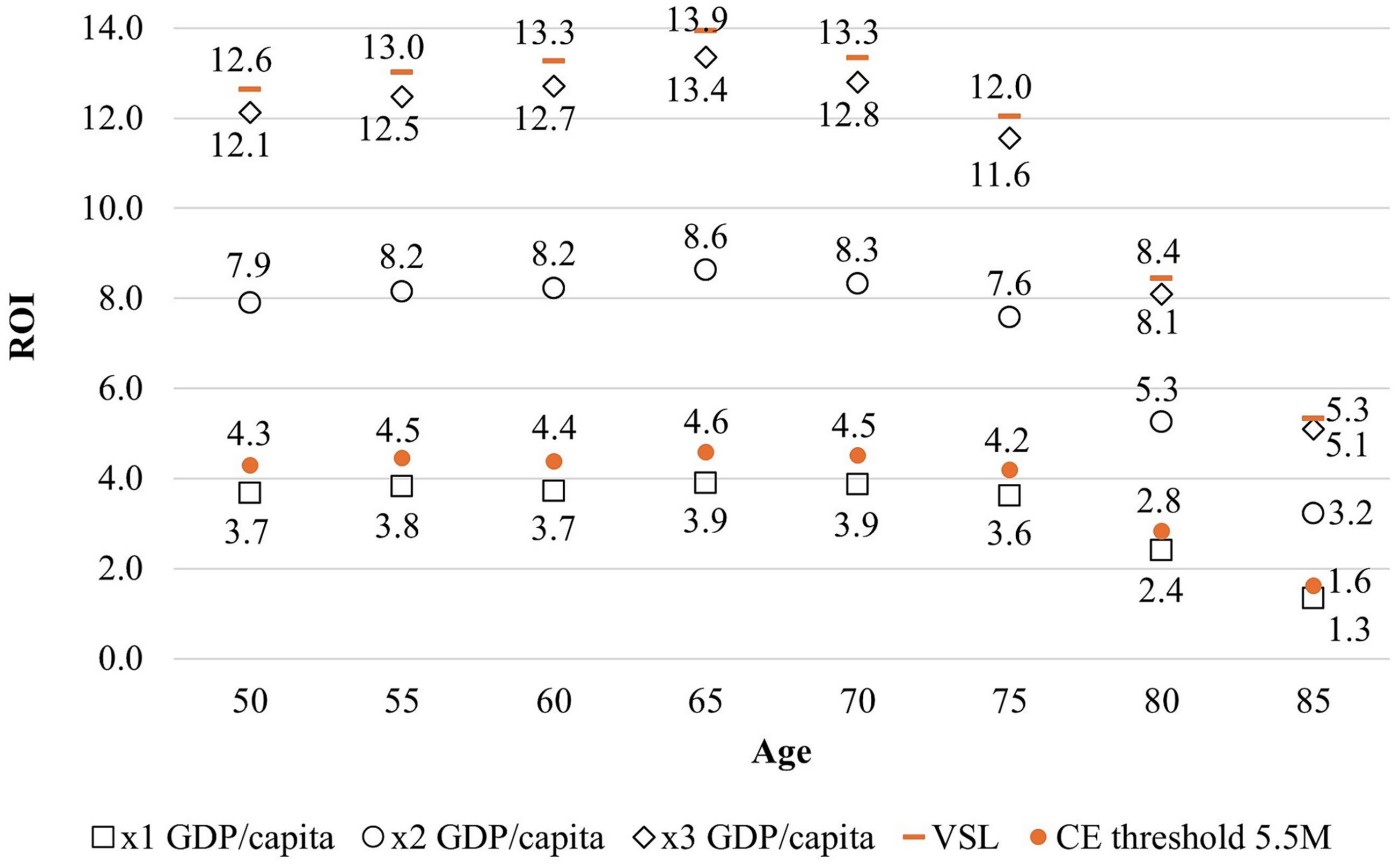
Scenario analyses for single cohorts (60, 65, 70, 75, 80, and 85 years) and high-risk cohorts (50 and 55 years). CE: cost-effectiveness; GDP: gross domestic product; M: million; QALYs: quality-adjusted life years; ROI: return on investment; VSL: value of statistical life.

Across all age groups of older adults aged ≥60 years and high-risk adults aged 50–59 years (Figure 2), the lowest societal ROIs were observed when the monetization of 1 QALY was based on 1 × GDP per capita. Societal ROIs increased when QALY valuations were based on the willingness-to-pay threshold of JPY 5.5 million per QALY, 2 × GDP per capita, and 3 × GDP per capita, reaching the highest societal ROIs when based on VSLY estimates of JPY 15 million.

#### 3.4.2 Financial analyses: multiple single cohorts

Across the single cohorts of older adults aged ≥60 years, the projected IRR ranged from 33 to 73%, with payback periods of 2.5–3.7 years (Table 2). For the high-risk single cohorts of adults aged 50 and 55 years, the projected IRR ranged from 66 to 68%, with payback periods of 2.7–2.8 years.

**Table 2:** Financial analyses: multiple single cohorts.

Age	50 (HR)	55 (HR)	60	65	70	75	80	85
IRR	68%	66%	56%	73%	62%	73%	52%	33%
Payback period (years)	2.7	2.8	3.2	2.6	2.9	2.5	3.0	3.7

HR: high-risk adults at increased risk of severe RSV disease due to certain underlying diseases; IRR: internal rate of return.

## 4 Discussion

Our study modeled a 5-year vaccination program with the adjuvanted RSVPreF3 vaccine, where vaccination was assumed to be administered as a single-dose to older adults aged ≥60 years and high-risk adults aged 50–59 years at increased risk of severe RSV disease due to certain underlying diseases. Overall, the results indicate that the modeled vaccination program was associated with substantial economic benefits over the 10-year analytic horizon. From the societal perspective, vaccination costs were offset by reductions in direct medical expenditures, monetized averted productivity losses, and QALY losses from avoided mortality and morbidity. By separately accounting for healthcare cost savings and monetized QALY losses, the analysis captured both direct resource utilization impacts and broader welfare effects of vaccination without overlap, thereby providing a more comprehensive societal perspective on value. Given Japan’s position among countries with the highest life expectancy, alongside government efforts to promote employment at older ages and a growing workforce aged ≥65 years, the evaluation of productivity-related benefits will become increasingly important going forward (48).

The projected ROIs were consistently higher than 1.0 with or without DWL considered, indicating that the projected economic benefits of vaccination more than doubled its associated costs. While gender and net immigration may influence baseline RSV incidence, these factors are not expected to materially affect aggregate vaccination impact estimates, and its exclusion from the model was therefore considered unlikely to influence the robustness of the results. The coverage rates adopted in the base case were based on reported age-specific coverage rates for the influenza vaccine in Japan. It should be noted that changes in coverage rates do not affect the ROI outcome, as both benefits and costs scale proportionally with coverage, thereby preserving the ratio. This proportionality is an inherent structural feature of the model, where non-linear effects were not incorporated. As a result, while coverage influences the absolute magnitude of costs and benefits, the relative relationship and ROI remain unchanged. Together, this suggests that the adjuvanted RSVPreF3 vaccination program in older adults is likely a beneficial use of scarce public funds, with the potential to deliver broad societal benefits that are large enough to offset vaccination costs. In the context of Japan’s rapidly aging population and rising healthcare expenditures, these findings are particularly relevant, as interventions with positive economic returns may contribute to the long-term sustainability of public finances. While the results suggest favorable societal returns associated with RSV vaccination, the findings should be interpreted within the context of the underlying model assumptions and key inputs used. Accordingly, future analyses incorporating more comprehensive real-world evidence and enhanced surveillance data, where available, may help to further refine these estimates.

DWL reflects the loss of economic efficacy and societal welfare that arises from market distortions (e.g., increased taxation to compensate for revenue losses caused by disease-related productivity reductions). In this analysis, ROIs were estimated both with and without the inclusion of DWL. Results indicated that the difference between these two scenarios was minimal among older adults aged ≥60 years, but more noticeable (approximately 0.1) among high-risk adults aged 50–59 years. This may be attributed to higher employment rates and productivity contribution among adults aged 50–59 years, where RSV-related productivity losses and associated tax fluctuations are more pronounced. Hence, these findings further underscore the necessity of promoting RSV vaccination among high-risk adults in their 50s, as vaccination can mitigate productivity losses, thereby offsetting DWL and enhancing the program’s value.

Results were found to be robust across a range of sensitivity analyses, with ROIs remaining above 0 across all ±20% parameter variations for both older adults aged ≥60 years and high-risk adults aged 50–59 years. While the valuation of one QALY based on a willingness-to-pay threshold of 1 × GDP per capita was the most influential parameter in both groups, ROIs remained favorable even at the –20% lower bound, reinforcing the substantial benefits of vaccination. Key epidemiological inputs such as RSV incidence also contributed to variability, underscoring the importance of accurate local RSV surveillance to better assess the vaccine’s impact in public health and economic domains.

Scenario analyses with alternative QALY valuations produced higher societal ROIs in both older adults aged ≥60 years and high-risk adults aged 50–59 years, compared to the base-case valuation of 1 × GDP per capita per QALY. This underscores the conservative nature of the base case analysis presented, suggesting that the broader societal benefits conferred by the adjuvanted RSVPreF3 vaccination may be greater than indicated by the base-case estimates. Importantly, the valuation of QALYs using VSL – a metric widely used in public policy to reflect how much society is willing to pay to reduce mortality risks – resulted in the highest ROIs. As VSL-based QALY valuations capture broader societal willingness to pay for health and risk reduction, this approach provided a more comprehensive measure of the societal benefits generated by adjuvanted RSVPreF3 vaccination in older adults. These findings suggest that the true economic and societal value of vaccination may be considerably greater than indicated by the base-case estimates, reinforcing the robustness of the study’s conclusions.

### 4.1 Comparisons with prior studies

To our knowledge, this is the first comprehensive CBA that applied an actuarial open population projection model and a Cobb–Douglas-based productivity framework to evaluate the broader economic value of RSV vaccination in Japan. By doing so, this study extends prior analyses by providing a fuller account of the economic externalities and cross-sectorial benefits of the adjuvanted RSVPreF3 vaccine among older adults aged ≥60 years and high-risk adults aged 50–59 years at increased risk for severe RSV disease.

To date, only two prior CBAs have assessed broader benefits of adult respiratory vaccinations with RSV included among the vaccines evaluated: the Office of Health Economics (OHE) analysis in the US and Germany (over a 40-year horizon), and the CBA by Theakston et al. in Japan (over various time horizons of 1-, 5-, 10-years, and lifetime). Consistent with our findings, both studies demonstrated that adult respiratory vaccination programs, including that of RSV, are likely to yield positive ROIs to society across multiple timeframes and settings (29, 30, 49). However, unlike earlier evaluations which followed static closed cohorts, our model combined an ILO-based dynamic actuarial population projection model with a full CBA framework that introduced new age cohorts annually, allowing demographic aging and labor-force dynamics to be reflected over time.

A further distinction lies in the valuation of QALY gains. The studies by OHE and Theakston et al. valued mortality using VSL/VSLY and incorporated morbidity-related QALY gains indirectly through cost-of-illness estimates, rather than by directly monetizing these gains. In contrast, the present analysis monetized QALY gains directly using GDP per capita in the base case scenario, thus capturing both morbidity- and mortality-related benefits within a single monetary framework. This approach is more comprehensive in directly valuing morbidity-related health gains, where the use of GDP per capita as the monetization basis is conservative relative to potentially higher cost-of-illness based estimations, allowing vaccination effects to be reflected consistently in the ROI estimates. Additionally, the current analysis modeled the impact of vaccination on productive output through the usage of a Cobb–Douglas function. This captured broader economic spillover effects and yielded more conservative estimates of productivity loss compared to the human-capital approach, that is based solely on wage-based productivity estimates. By translating productivity gains into associated tax revenue and quantifying the resulting DWL, the present framework captured societal benefits of vaccination that are not reflected in QALY-based valuations alone. These methodological differences collectively illustrate the influence of input choices and model structure on economic estimates, providing a complementary perspective that may better reflect fiscal and labor-market implications of RSV vaccination. Similar methodological approaches have been applied in other vaccination studies (particularly for pediatric immunization programs) (50, 51), further demonstrating the utility of broad economic frameworks in capturing vaccine-mediated benefits.

### 4.2 Strengths and limitations

A key strength of this study is the use of actuarial population projections to enable open population projection modeling. By accounting for age-specific mortality and demographic changes over time, this approach more accurately reflects real-world population shifts, including aging and longevity trends (52). Such modeling improves the validity of long-term forecasts for estimating a vaccines’ costs and benefits, ensuring that the results remain relevant for policy and planning purposes by stakeholders.

Another strength of this analysis is the use of the Cobb–Douglas production function to translate work-hour losses into GDP loss. Unlike the conventional human capital approach, which assumes a full one-to-one loss of production per work hour lost, the Cobb–Douglas production function captures the compensating role of capital in maintaining output, thereby providing a more realistic estimate of productivity costs.

Additionally, DWL estimation was integrated in this analysis to account for welfare costs from financing and market distortions. To our knowledge, this represents the first application of DWL within a vaccine health economic evaluation, enabling a more robust assessment of the vaccine’s societal value and providing further insights into strategies that maximize public welfare.

Limitations to this analysis should be acknowledged. The disease burden estimates in the present study were approximated using published scientific literature relevant to the Japanese setting and were considered the most suitable available inputs in the absence of comprehensive national surveillance data. Nevertheless, the epidemiological inputs used relied on limited observational data and proxy measures for RSV, which may under- or over-estimate the true disease burden in Japanese adults. In addition, limited routine RSV testing in older adults and the absence of comprehensive national surveillance for RSV in Japan may reduce the accuracy of reported incidence and prevalence, potentially leading to underestimation of the true disease burden (23). As the reliance on proxy indicators may not fully capture regional variability and temporal trends in RSV epidemiology, model estimates may not reflect the most current epidemiological patterns in Japan. Although sensitivity analyses demonstrated that our findings remained robust across plausible ranges of key epidemiological parameters, these estimates should not be regarded as definitive for policy decision-making. The findings of this analysis should therefore be interpreted in the context of uncertainties surrounding the accuracy of the epidemiological inputs used. This highlights the need for strengthened RSV surveillance and improved real-world data collection in Japan, to reduce parameter uncertainty and support more robust future economic evaluations.

Secondly, potential overlap in comorbidities within the high-risk population aged 50–59 years could not be formally accounted for, due to the absence of granular co-occurrence data in available Japanese databases. Hence, the size of the target population may be marginally overestimated to the extent that such overlap exists. This is an area for refinement in future analyses, as more detailed epidemiological data becomes available.

Thirdly, macroeconomic impacts were estimated using a stylized Cobb–Douglas framework and represent indicative productivity effects rather than observed outcomes. Similarly, monetized health gains reflect standard valuation approaches and should be interpreted as approximations subject to uncertainty and contextual variation.

Lastly, the marginal costs of increasing labor tax used in the calculation of DWL in Japan was assumed to be equivalent to that in the EU to facilitate cross-country comparison with similar analyses conducted in EU settings (53), although a Japan-specific estimate (0.22) was identified in the literature (54). Hence, country-specific differences in tax structures and labor market responses may lead to variation in the true magnitude of DWL in Japan. Nonetheless, a feasibility analysis comparing the Japan-specific value of 0.22 against the EU-derived estimate of 0.79 showed minimal impact on overall ROI results. Specifically, using the Japan-specific value, ROI (with DWL) remained at 3.3 among older adults aged ≥60 years and 3.8 among high-risk adults aged 50–59 years. Therefore, the EU-based parameter was retained in the base case as a conservative assumption.

## 5 Conclusion

This study showed that an adjuvanted RSVPreF3 vaccination program against RSV implemented over a period of 5 years for Japanese older adults, including high-risk adults aged 50–59 years at increased risk for severe RSV disease and older adults aged ≥60 years, is associated with a favorable societal ROI. This suggests that from a societal perspective, vaccination of older adults is good value for money and consistently produces positive returns, where vaccine acquisition and administration costs are expected to be offset by broader benefits.

These findings provide supportive evidence for health policy decision-making regarding vaccination with the adjuvanted RSVPreF3 vaccine in Japan, where efforts to increase the adjuvanted RSVPreF3 vaccine coverage in high-risk adults aged 50–59 years at increased risk for severe RSV disease and older adults aged ≥60 years may be an economically feasible and sustainable use of public funds.

## Data Availability

The datasets generated and/or analyzed during the current study are available from the corresponding author on reasonable request.
