# Supplementary material for: Unlocking the value of RSV vaccination in Japan: a return on investment analysis using an integrated actuarial-macroeconomic model
Source: Front Public Health. 2026 Aug 4;14:1871916. doi: 10.3389/fpubh.2026.1871916 (PMC13481221; doi:10.3389/fpubh.2026.1871916)
Supplement: Supplementary file 1 [file Data_sheet_1.docx]

Supplementary Material

Unlocking the Value of RSV Vaccination in Japan: A Return on Investment Analysis Using an Integrated Actuarial-Macroeconomic Model

Eleftherios Zarkadoulas^1*^, Asako Akimoto^2^, Deven Chauhan^3^, Yufan Ho^4^, Christianah Kolajo^5^, Nikolaos Kotsopoulos^6,7^, Kentaro Tajima^2^

^1^GSK, Wavre, Belgium

^2^GSK, Tokyo, Japan

^3^GSK, London, United Kingdom

^4^GSK, Singapore, Singapore

^5^GSK, Rockville, Maryland, United States

^6^Health Economics, Global Market Access Solutions Sàrl, Chardonne, Switzerland

^7^University of Athens, Athens, Greece

*Correspondence:
Eleftherios Zarkadoulas
[eleftherios.x.zarkadoulas@gsk.com](mailto:eleftherios.x.zarkadoulas@gsk.com)

# Supplementary Table S1. Epidemiological inputs

| **Parameter by Input & Age** | **Value** | **Description** | **Source** |
| --- | --- | --- | --- |
| **Case fatality rate**  **(hospitalized), %** |  |  |  |
| 50–54 years | 3.7 | Ratio of mortality rate to hospitalization rate | (1-10) |
| 55–59 years | 4.1 |  |  |
| 60–64 years | 2.7 |  |  |
| 65–69 years | 7.0 |  |  |
| 70–74 years | 7.0 |  |  |
| 75–79 years | 4.2 |  |  |
| 80–84 years | 4.2 |  |  |
| 85–89 years | 4.2 |  |  |
| 90–100 years | 4.2 |  |  |
| **RSV incidence rate, %** |  |  |  |
| 50–54 years | 4.9 | Age-specific incidence of RSV | (1, 11-14) |
| 55–59 years | 4.9 |  |  |
| 60–64 years | 4.2 |  |  |
| 65–69 years | 4.2 |  |  |
| 70–74 years | 4.2 |  |  |
| 75–79 years | 4.2 |  |  |
| 80–109 years | 4.2 |  |  |
| **LRTD rate, %** |  |  |  |
| 50–54 years | 60.7 | Rate of RSV cases leading to LRTD | (1, 3, 4) |
| 55–59 years | 60.7 |  |  |
| 60–64 years | 50.2 |  |  |
| 65–69 years | 50.2 |  |  |
| 70–74 years | 50.2 |  |  |
| 75–79 years | 50.2 |  |  |
| 80–109 years | 50.2 |  |  |
| **Abbreviations:** LRTD: lower respiratory tract disease; RSV: respiratory syncytial virus. | | | |

# Supplementary Table S2. Vaccine inputs

| **Parameter by Input & Age** | **Value** | **Description** | **Source** |
| --- | --- | --- | --- |
| **Coverage rate, %** |  |  |  |
| 50–54 years | 45.2 | Age-specific vaccination coverage rate | (1, 15) |
| 55–59 years | 46.9 |  |  |
| 60–64 years | 52.4 |  |  |
| 65–69 years | 57.5 |  |  |
| 70–74 years | 65.7 |  |  |
| 75–79 years | 65.7 |  |  |
| 80–109 years | 65.7 |  |  |
| **Vaccination costs, JPY** |  |  |  |
| Vaccination costs per dose | 20,000.00 | Acquisition cost per dose | (1) |
| Administration vaccination costs | 3,310.00 | Administration cost per dose | (1) |
| **Abbreviations:** JPY: Japanese Yen.  **Note:** Monetary values appear in JPY, 2024. | | | |

# Supplementary Table S3. HCRU, unit prices, and direct medical costs

| **Parameter by Input & Age** | **Value** | **Description** | **Source** |
| --- | --- | --- | --- |
| **LRTD outpatient rate, number of outpatient visits per RSV case** | | | |
| 50–54 years | 1.237 | Rate of LRTD cases using outpatient services | (1) |
| 55–59 years | 1.237 |  |  |
| 60–64 years | 0.840 |  |  |
| 65–69 years | 0.840 |  |  |
| 70–74 years | 0.840 |  |  |
| 75–79 years | 0.655 |  |  |
| 80–109 years | 0.655 |  |  |
| **LRTD hospitalization rate, number of hospitalizations per RSV case** | | | |
| 50–54 years | 0.096 | Rate of LRTD cases hospitalized | (1) |
| 55–59 years | 0.096 |  |  |
| 60–64 years | 0.111 |  |  |
| 65–69 years | 0.111 |  |  |
| 70–74 years | 0.111 |  |  |
| 75–79 years | 0.325 |  |  |
| 80–109 years | 0.325 |  |  |
| **URTD outpatient rate, number of outpatient visits per RSV case** | | | |
| 50–54 years | 0.791 | Rate of LRTD cases using outpatient services | (1) |
| 55–59 years | 0.791 |  |  |
| 60–64 years | 0.452 |  |  |
| 65–69 years | 0.452 |  |  |
| 70–74 years | 0.452 |  |  |
| 75–79 years | 0.554 |  |  |
| 80–109 years | 0.554 |  |  |
| **LRTD outpatient costs, JPY** | | | |
| 50–54 years | 15,102.18 | Unit cost applied to each outpatient case | (1) |
| 55–59 years | 15,102.18 |  |  |
| 60–64 years | 21,173.07 |  |  |
| 65–69 years | 21,173.07 |  |  |
| 70–74 years | 21,173.07 |  |  |
| 75–79 years | 128,764.72 |  |  |
| 80–109 years | 128,764.72 |  |  |
| **URTD outpatient costs, JPY** | | | |
| 50–54 years | 6,059.50 | Unit cost applied to each outpatient case | (1) |
| 55–59 years | 6,059.50 |  |  |
| 60–64 years | 4,580.00 |  |  |
| 65–69 years | 4,580.00 |  |  |
| 70–74 years | 4,580.00 |  |  |
| 75–79 years | 6,536.00 |  |  |
| 80–109 years | 6,536.00 |  |  |
| **Abbreviations:** HCRU: healthcare resource utilization; JPY: Japanese Yen; LRTD: lower respiratory tract disease; RSV: respiratory syncytial virus; URTD: upper respiratory tract disease.  **Note:** Aggregate model assigns single cost estimates to patients. Monetary values appear in JPY, 2024. | | | |

# Supplementary Table S4. HRQoL and baseline utility

| **Parameter by Outcome & Age** | **Value** | **Description** | **Source** |
| --- | --- | --- | --- |
| **Unvaccinated URTD QALY loss** |  |  |  |
| 50–54 years | 0.018 | QALY loss by event | (1) |
| 55–59 years | 0.018 |  |  |
| 60–64 years | 0.018 |  |  |
| 65–69 years | 0.018 |  |  |
| 70–74 years | 0.018 |  |  |
| 75–79 years | 0.018 |  |  |
| 80–84 years | 0.018 |  |  |
| 85–100 years | 0.018 |  |  |
| **Unvaccinated LRTD QALY loss** |  |  |  |
| 50–54 years | 0.060 | QALY loss by event | (1) |
| 55–59 years | 0.060 |  |  |
| 60–64 years | 0.060 |  |  |
| 65–69 years | 0.060 |  |  |
| 70–74 years | 0.060 |  |  |
| 75–79 years | 0.060 |  |  |
| 80–84 years | 0.060 |  |  |
| 85–100 years | 0.060 |  |  |
| **Vaccinated URTD QALY loss** |  |  |  |
| 50–54 years | 0.018 | QALY loss by event | (1) |
| 55–59 years | 0.018 |  |  |
| 60–64 years | 0.018 |  |  |
| 65–69 years | 0.018 |  |  |
| 70–74 years | 0.018 |  |  |
| 75–79 years | 0.018 |  |  |
| 80–84 years | 0.018 |  |  |
| 85–100 years | 0.018 |  |  |
| **Vaccinated LRTD QALY loss** |  |  |  |
| 50–54 years | 0.060 | QALY loss by event | (1) |
| 55–59 years | 0.060 |  |  |
| 60–64 years | 0.060 |  |  |
| 65–69 years | 0.060 |  |  |
| 70–74 years | 0.060 |  |  |
| 75–79 years | 0.060 |  |  |
| 80–84 years | 0.060 |  |  |
| 85–100 years | 0.060 |  |  |
| **QALY loss from AE** |  |  |  |
| Vaccinated loss from AE 50–100 years | <0.001 | QALY loss by AE | (1) |
| **Abbreviations:** AE: adverse events; HRQoL: health-related quality of life; LRTD: lower respiratory tract disease; QALY: quality-adjusted life years; URTD: upper respiratory tract disease. | | | |

# Supplementary Table S5. Demographical and actuarial inputs

| **Parameter by Age** | **2024–2029** | **2030–2039** | **2040–2049** | **2050–2059** | **2060–2069** | **2070+** | **Source** |
| --- | --- | --- | --- | --- | --- | --- | --- |
| **Labor force participation rate, %** | | | | | | | |
| 50–54 years | 88.20 | 90.80 | 90.80 | 90.80 | 90.80 | 90.80 | (16, 17) |
| 55–59 years | 85.30 | 90.42 | 90.42 | 90.42 | 90.42 | 90.42 |  |
| 60–64 years | 76.40 | 88.76 | 88.76 | 88.76 | 88.76 | 88.76 |  |
| 65+ years | 26.10 | 31.07 | 31.07 | 31.07 | 31.07 | 31.07 |  |
| **Unemployment rate, %** | | | | | | | |
| 50–54 years | 2.20 | 2.20 | 2.20 | 2.20 | 2.20 | 2.20 | (16) |
| 55–59 years | 2.40 | 2.40 | 2.40 | 2.40 | 2.40 | 2.40 |  |
| 60–64 years | 2.80 | 2.80 | 2.80 | 2.80 | 2.80 | 2.80 |  |
| 65+ years | 0.00 | 0.00 | 0.00 | 0.00 | 0.00 | 0.00 |  |
| **Disability rate** | | | | | | | |
| 50–54 years | 0.06 | 0.06 | 0.06 | 0.06 | 0.06 | 0.06 | (18) |
| 55–59 years | 0.06 | 0.06 | 0.06 | 0.06 | 0.06 | 0.06 |  |
| 60–64 years | 0.11 | 0.11 | 0.11 | 0.11 | 0.11 | 0.11 |  |
| 65+ years | 0.11 | 0.11 | 0.11 | 0.11 | 0.11 | 0.11 |  |
| **Average labor market exit age, years** | 65 | 65 | 65 | 65 | 65 | 65 | (19) |

# Supplementary Table S6. Broader economic costs

| **Variable** | **Value** | **Description** | **Source** |
| --- | --- | --- | --- |
| Workhours worked per year (average), hours | 1719 | Mean number of hours worked per year | (20) |
| Growth in labor hours worked, % |  |  |  |
| 2024–2031 | -0.52 | Annual change in workhours worked by time period | (17) |
| 2032–2041 | -0.52 |  |  |
| 2042–2051 | -0.52 |  |  |
| 2052–2061 | -0.52 |  |  |
| 2062–2071 | -0.52 |  |  |
| 2072+ | -0.52 |  |  |
| Duration of vaccination, years | 5.00 |  | Assumption |
| Time horizon, years | 10.00 |  | Assumption |
| Cobb-Douglas parameter | 0.50 |  | (21) |
| Social security contributions | 0.133 | Fraction of GDP contributions | (22) |
| Corporate income tax | 0.045 | Fraction of GDP paid as corporate tax |  |
| Personal income tax | 0.064 | Fraction of GDP paid as income tax |  |
| Value added tax | 0.071 | Fraction of GDP paid as consumption tax |  |
| Wage growth | 0.051 | Nominal wage growth per year |  |
| Consumer Price Index | 0.027 | Annual cost inflation |  |
| Discount rate | 0.02 | Discounting rate | (1) |
| GDP (trillion), JPY | 607.41 | GDP in market prices | (17, 23) |
| GDP growth, % |  |  | (24) |
| 2025 | 3.4 | Annual growth of nominal GDP for the specified period | (17, 23) |
| 2026 | 2.9 |  | (24) |
| 2027 | 2.9 |  |  |
| 2028 | 3.0 |  |  |
| 2029 | 3.0 |  |  |
| 2030–2035 | 3.1 |  |  |
| 2036–2041 | 3.0 |  |  |
| 2042–2051 | 3.1 |  |  |
| 2052–2061 | 3.2 |  |  |
| 2062+ | 3.3 |  |  |
| URTD outpatient workhour loss |  |  |  |
| 18–59 years | 9.47 | 1 doctor visit per outpatient event | (1) |
| 60–74 years | 5.42 |  |  |
| 75+ years | 6.64 |  |  |
| LRTD outpatient workhour loss |  |  |  |
| 18–59 years | 13.41 |  |  |
| 60–74 years | 8.96 |  |  |
| 75+ years | 5.31 |  |  |
| LRTD hospitalizations workhour loss |  |  |  |
| 15–59 years | 16.16 | Work hour loss per event, 30 days length of stay | (1) |
| 60–74 years | 21.86 |  |  |
| 75+ years | 123.98 |  |  |
| LRTD ICU workhour loss | 0.00 | Assuming 22 workdays per month | (1) |
| Value of 1 QALY (base case), JPY | 4.8M | GDP per capita x1 | (25) |
| Value of 1 QALY (option 1), JPY | 4.8M | Willingness to pay for 1 QALY | Assumption |
| Value of 1 QALY (option 2), JPY | 9.6M | GDP per capita x2 | Calculation |
| Value of 1 QALY (option 3), JPY | 14.4M | GDP per capita x3 | Calculation |
| DWL factor | 0.79 | Cost of raising 1 monetary unit of tax revenue | (26) |
| **Abbreviations:** CPI: consumer price index; DWL: deadweight loss; GDP: gross domestic product; ICU: intensive care unit; JPY: Japanese Yen; LRTD: lower respiratory tract disease; URTD: upper respiratory tract disease.  **Note:** Monetary values appear in JPY, 2024. | | | |

# References

1.	Tajima K, Zarkadoulas E, Simons J, Kurai D, Grace M, Graham J, et al. Cost-effectiveness of the adjuvanted respiratory syncytial virus prefusion F protein (RSVPreF3) vaccine in Japanese adults aged 50-59 years at increased risk of severe RSV disease and those aged ≥60 years. Expert Rev Vaccines. 2026;25(1):2626921.

2.	Nguyen-Van-Tam JS, O'Leary M, Martin ET, Heijnen E, Callendret B, Fleischhackl R, et al. Burden of respiratory syncytial virus infection in older and high-risk adults: a systematic review and meta-analysis of the evidence from developed countries. Eur Respir Rev. 2022;31(166).

3.	Ison MG, Papi A, Athan E, Feldman RG, Langley JM, Lee DG, et al. Efficacy, safety, and immunogenicity of the AS01(E)-adjuvanted respiratory syncytial virus prefusion F protein vaccine (RSVPreF3 OA) in older adults over three respiratory syncytial virus seasons (AReSVi-006): a multicentre, randomised, observer-blinded, placebo-controlled, phase 3 trial. Lancet Respir Med. 2025;13(6):517-29.

4.	GSK. Data on file (AReSvi-006 phase 3 clinical trial). 2024.

5.	Katsurada N, Suzuki M, Aoshima M, Yaegashi M, Ishifuji T, Asoh N, et al. The impact of virus infections on pneumonia mortality is complex in adults: a prospective multicentre observational study. BMC Infect Dis. 2017;17(1):755.

6.	Tsuzuki S, Yoshihara K. The characteristics of influenza-like illness management in Japan. BMC Public Health. 2020;20(1):568.

7.	Zhang Q, Feng S, Wong IOL, Ip DKM, Cowling BJ, Lau EHY. A population-based study on healthcare-seeking behaviour of persons with symptoms of respiratory and gastrointestinal-related infections in Hong Kong. BMC Public Health. 2020;20(1):402.

8.	Molnar D, La E, Verelst F, et al. Modeling the clinical and economic burden of respiratory syncytial virus (RSV) among adults ≥60 years of age in the United States. In: Poster presented at: Respiratory Synctial Virus Foundation (RSVVW) 2023 (7th ReSViNET Conference); Lisbon, Portugal; 2023 Feb 22–24.

9.	e-stat. Confirmed number nationwide viewing table 37 total number of patients, sex and age group (5 years old) × subclassification of injuries and diseases. 2023. Available from: <https://www.e-stat.go.jp/dbview?sid=0004002503>. Last accessed: May 2026

10.	e-stat. Statistics by social medical treatment procedures (formerly: Survey by social medical treatment procedures) for 2023, Reiwa 5th. Tables 14–1 (for age <75, inpatient), 14–2 (age 75+, inpatient), 15–1 (age <75, outpatient), 15–2 (age 75+ outpatient) 2024. Available from: <https://www.e-stat.go.jp/stat-search/files?page=1&layout=datalist&toukei=00450048&tstat=000001029602&cycle=7&tclass1=000001217700&tclass2=000001217701&tclass3=000001217760&cycle_facet=tclass1%3Atclass2%3Atclass3&tclass4val=0>. Last accessed: May 2026

11.	Kurai D, Natori M, Yamada M, Zheng R, Saito Y, Takahashi H. Occurrence and disease burden of respiratory syncytial virus and other respiratory pathogens in adults aged≥ 65 years in community: a prospective cohort study in Japan. Influenza Other Respir Viruses. 2022;16(2):298-307.

12.	Li Y, Kulkarni D, Begier E, Wahi-Singh P, Wahi-Singh B, Gessner B, et al. Adjusting for Case Under-Ascertainment in Estimating RSV Hospitalisation Burden of Older Adults in High-Income Countries: a Systematic Review and Modelling Study. Infect Dis Ther. 2023;12(4):1137-49.

13.	McLaughlin JM, Khan F, Begier E, Swerdlow DL, Jodar L, Falsey AR. Rates of Medically Attended RSV Among US Adults: A Systematic Review and Meta-analysis. Open Forum Infect Dis. 2022;9(7):ofac300.

14.	Falsey AR, Hennessey PA, Formica MA, Cox C, Walsh EE. Respiratory syncytial virus infection in elderly and high-risk adults. N Engl J Med. 2005;352(17):1749-59.

15.	National Institute of Infectious Diseases. Infectious agents surveillance report (IASR), Vol. 44, No. 11 (No. 525), November 2023. 2023. Available from: <https://id-info.jihs.go.jp/surveillance/iasr/44/525/525.pdf>. Last accessed: May 2026

16.	e-stat. Table I-B-I Population aged 15 years old and over by labour force status, status in employment, type of employment, duration of employment contract, agriculture and forestry or non-agricultural industries, number of persons engaged in enterprise and age group. 2023. Available from: <https://www.e-stat.go.jp/en/stat-search/files?page=1&layout=datalist&toukei=00200531&tstat=000001226891&cycle=7&year=20240&month=0&tclass1=000001227003&result_back=1&cycle_facet=tclass1&tclass2val=0>. Last accessed: April 2026

17.	OECD. (DF_EO114_LTB) Economic outlook no 114 - December 2023 - long-term baseline projections 2024. Available from: [https://data-explorer.oecd.org/vis?lc=en&df[ds]=DisseminateArchiveDMZ&df[id]=DF_EO114_LTB&df[ag]=OECD&df[vs]=&av=true&dq=JPN.GDPVD%2BERS1574%2BETPT%2BPOP%2BPOPS%2BGDP.S0.A&to[TIME_PERIOD]=false&vw=tb&lb=bt&pg=0&pd=2024%2C2050](https://data-explorer.oecd.org/vis?lc=en&df%5bds%5d=DisseminateArchiveDMZ&df%5bid%5d=DF_EO114_LTB&df%5bag%5d=OECD&df%5bvs%5d=&av=true&dq=JPN.GDPVD%2BERS1574%2BETPT%2BPOP%2BPOPS%2BGDP.S0.A&to%5bTIME_PERIOD%5d=false&vw=tb&lb=bt&pg=0&pd=2024%2C2050). Last accessed: April 2026

18.	Saito T, Imahashi K, Yamaki C. The first use of the Washington Group Short Set in a national survey of Japan: characteristics of the new disability measure in comparison to an existing disability measure. International Journal of Environmental Research and Public Health. 2024;21(12):1643.

19.	Service JP. Eligibility requirements, payment start date, and pension amount for basic old-age pension 2025. Available from: <https://www.nenkin.go.jp/service/jukyu/seido/roureinenkin/jukyu-yoken/20150401-02.html>. Last accessed: January 2026

20.	The Japan Institute for Labor Policy and Training. Table 6-1: Average annual hours actually worked per person in employment 2023. Available from: <https://www.jil.go.jp/kokunai/statistics/databook/2024/06/d2024_6T-01.pdf>. Last accessed: January 2026

21.	Hirakata N, Koike Y. The labor share, capital-labor substitution, and factor augmenting technologies: Bank of Japan; 2018. Available from: <https://www.boj.or.jp/en/research/wps_rev/wps_2018/data/wp18e20.pdf>. Last accessed: January 2026

22.	OECD. Revenue statistics 2024. Available from: [https://data-explorer.oecd.org/vis?lc=en&tm=revenue%20statistics&pg=0&snb=247&vw=tb&df[ds]=dsDisseminateFinalDMZ&df[id]=DSD_REV_COMP_GLOBAL%40DF_RSGLOBAL&df[ag]=OECD.CTP.TPS&df[vs]=2.1&dq=JPN..S13.T_2000..PT_B1GQ.A&lom=LASTNPERIODS&lo=10&to[TIME_PERIOD]=false](https://data-explorer.oecd.org/vis?lc=en&tm=revenue%20statistics&pg=0&snb=247&vw=tb&df%5bds%5d=dsDisseminateFinalDMZ&df%5bid%5d=DSD_REV_COMP_GLOBAL%40DF_RSGLOBAL&df%5bag%5d=OECD.CTP.TPS&df%5bvs%5d=2.1&dq=JPN..S13.T_2000..PT_B1GQ.A&lom=LASTNPERIODS&lo=10&to%5bTIME_PERIOD%5d=false). Last accessed: April 2026

23.	Cabinet Office. 2025. Available from: <https://www.cao.go.jp/index-e.html>. Last accessed: July 2025

24.	World Bank. GDP growth (annual %) - Japan 2025. Available from: <https://data.worldbank.org/indicator/NY.GDP.MKTP.KD.ZG?locations=JP>. Last accessed: January 2026

25.	The Japan Institute for Labor Policy and Training. Databook of international labour statistics 2025. Available from: <https://www.jil.go.jp/kokunai/statistics/databook/2025/documents/Databook2025.pdf>. Last accessed: January 2026

26.	Barrios S, Pycroft J, Saveyn B. The marginal cost of public funds in the EU: the case of labour versus green taxes. Directorate General Taxation and Customs Union, European Commission; 2013.
